# Particulate air pollution and chronic ischemic heart disease in the eastern United States: a county level ecological study using satellite aerosol data

**DOI:** 10.1186/1476-069X-8-26

**Published:** 2009-06-12

**Authors:** Zhiyong Hu, K Ranga Rao

**Affiliations:** 1 Department of Environmental Studies, University of West Florida, Pensacola, Florida, USA; 2 Center for Environmental Diagnostics and Bioremediation, University of West Florida, Pensacola, Florida, USA

## Abstract

**Background:**

There are several known factors that cause ischemic heart disease. However, the part played by air pollution still remains something of a mystery. Recent attention has focused on the chronic effect of particulate matter on heart disease. Satellite-derived aerosol optical depth (AOD) was found to be correlated with *PM*_2.5 _in the eastern US. The objective of this study was to examine if there is an association between aerosol air pollution as indicated by AOD and chronic ischemic heart disease (CIHD) in the eastern US.

**Methods:**

An ecological geographic study method was employed. Race and age standardized mortality rate (SMR) of CIHD was computed for each of the 2306 counties for the time period 2003–2004. A mean AOD raster grid for the same period was derived from Moderate Resolution Imaging Spectrometer (MODIS) aerosol data and the average AOD was calculated for each county. A bivariate Moran's I scatter plot, a map of local indicator of spatial association (LISA) clusters, and three regression models (ordinary least square, spatial lag, and spatial error) were used to analyze the relationship between AOD and CIHD SMR.

**Results:**

The global Moran's I value is 0.2673 (*p *= 0.001), indicating an overall positive spatial correlation of CIHD SMR and AOD. The entire study area is dominated by spatial clusters of AOD against SMR (high AOD and high SMR in the east, and low AOD and low SMR in the west) (permutations = 999, *p *= 0.05). Of the three regression models, the spatial error model achieved the best fit (R^2 ^= 0.28). The effect of AOD is positive and significant (beta = 0.7774, p = 0.01).

**Conclusion:**

Aerosol particle pollution has adverse effect on CIHD mortality risk in the eastern US. High risk of CIHD mortality was found in areas with elevated levels of outdoor aerosol air pollution as indicated by satellite derived AOD. The evidence of the association would support targeting of policy interventions on such areas to reduce air pollution levels. Remote sensing AOD data could be used as an alternative health-related indictor of air quality.

## Background

Ischemic heart disease (IHD), also called coronary artery disease or coronary heart disease, is a condition that the blood vessels are narrowed or blocked due to the deposition of cholesterol plaques on their walls. This affects the supply of blood to the heart and reduces the supply of oxygen and nutrients to the heart musculature. This may eventually result in a portion of the heart being suddenly deprived of its blood supply leading to the death of that area of heart tissue, resulting in a heart attack. IHD, with its associated symptoms, presents a major public health challenge. It is well known that IHD risk increases with age, smoking, cholesterol level, blood pressure and the disease is more common in men, diabetes patients and those who have close relatives with ischemic heart disease. The part played by the external environment, however, still remains something of a mystery.

Numerous epidemiological studies have indicated that exposure to fine particulate matter (particles smaller than 2.5 micrometers, *PM*_2.5_) is associated with asthma, respiratory infections, lung cancer, cardiovascular problems, and premature death [1-5]. A few have examined coronary heart disease, finding evidence for acute effects on mortality and hospital admissions [6-8]. Recently, attention has focused on whether there is an association between chronic exposure to air pollution and coronary heart disease [9]. In an ecological study at the census enumeration district level, Maheswaran et al. [9] found an association between nitrogen oxides and, to a lesser extent, particulate matter (PM_10_) and carbon monoxide, and coronary heart disease mortality in Sheffield, UK.

Typically, air pollution epidemiological studies rely on ambient observations from sparse monitoring networks to provide metrics of exposure, as in studies of PM and cardiovascular diseases [2,4,9-12]. Methods of exposure assessment include averaging multiple monitors within each enumeration unit or study site [4,10,11], assigning the exposure value of the nearest monitor to each case/control [2,12] and spatial interpolation/modelling method [9]. Ground monitoring data often lacks spatially complete coverage. Assessment of the exposure to air pollution using in situ observations is hampered by the difficulties in the highly variable concentrations in space and time. Ground monitors are rare in rural areas and temporal air quality estimates for particulates can vary on an hourly to weekly basis. Public health concerns compel efforts to broaden spatial and temporal coverage. The repetitive and broad-area coverage of satellites may allow atmospheric remote sensing to offer a unique opportunity to monitor air quality at continental, national and regional scales. Studies have found correlations between satellite aerosol optical depth (AOD), which describes the mass of aerosols in an atmospheric column, and PM_2.5 _ground concentration measurements in the eastern and mid-west USA [13-17] and other land parts of the world [18-20]. Satellite AOD could be used as an alternative indicator of air quality in air pollution epidemiological studies.

The MODIS (Moderate Resolution Imaging Spectrometer) instrument flies on polar-orbiting and sun-synchronous Terra and Aqua satellites of the Earth Observation System (EOS). MODIS performs measurements in the solar to thermal infrared spectrum region from 0.410 to 14.235 *μ*m. The MODIS sensor was expected to be the key for monitoring global aerosol properties. Not only have MODIS aerosol products been used to answer scientific questions about radiation and climate, they are being used for applications not previously intended. Some examples include monitoring surface air quality for health [15,16,21-24]. In our recent study [17,25] using the year 2004 data, we spatio-temporally collated daily MODIS AOD and PM_2.5 _mass concentration monitored by US Environmental Protection Agency (EPA)'s Air Quality System (AQS). Pearson's correlation analysis and geographically weighted regression (GWR) were conducted. We found significant positive correlations between PM_2.5 _and AOD to the east of the -100° longitude line during warm months (April-September). Eighteen of the twenty sites with highest correlation values (r > 0.8, p = 0.05) are in the east. The average correlation is 0.67 in the east and 0.22 in the west. GWR predicts well in the eastern US and poorly in the west, as indicated by a map of local R square. The coefficient raster surface for AOD exhibits regional variation. The relationship between PM_2.5 _and AOD is not spatially consistent (stationary) across the conterminous states. Eastern US shows higher AOD coefficient values, while values in the west are lower. Since there exists correlation between PM_2.5 _and MODIS AOD in the eastern US, if we could make a hypothesis that PM_2.5 _has adverse effect on CIHD, then we could also hypothesize that there is an association between MODIS AOD and CIHD.

The objective of this study was to examine if there is an association between particulate air pollution and chronic ischemic heart disease (CIHD). The study adopted an ecological method using aggregate disease mortality data at the county level for the eastern United States and MODIS derived AOD data. Exploratory spatial analysis methods and regression models were used to link CIHD mortality rate with satellite AOD.

## Methods

### MODIS data

One of the fundamental aerosol products from MODIS is spectral AOD (or *τ*). The MODIS level 2 files are produced everyday at the spatial resolution of a 10 ×10 1-km (at nadir)-pixel array and represent the first level of MODIS aerosol retrieval. The latest version of the MODIS aerosol retrieval algorithm is Collection 5 (C005) [26]. The aerosol retrieval makes use of blue, red, and thermal infrared spectral channels and a number of other bands to help with cloud rejection and other screening procedures. The aerosol algorithm relies on calibrated, geolocated reflectance data (known as 'Level 1B'). In addition, the MODIS algorithm uses cloud mask product [27], atmospheric profile product and ancillary data from NCEP (National Center for Environmental Prediction) analyses, including hourly meteorological analysis and daily ozone analysis. Different dynamic aerosol models (biomass burning, dust aerosol, and aerosol from industrial/urban origin) are used to determine the aerosol optical properties used in the algorithm for different parts of the land. In general, the highest correlations between AOD and PM_2.5 _are found in regions where the algorithm employs the sulfate aerosol type model (urban/industrial model) [22]. Over the land of the conterminous US, the aerosol retrieval algorithm used the urban/industrial aerosol model for the east and the dust/biomass burning model for the west. The splitting line is at approximately -100° longitude. Industrial/urban aerosols are created by the burning of coal and fossil fuel and are considered as the main anthropogenic sources of aerosols which impact human health.

For the study, daily level 2 MODIS raster data layers (2003–2004) were obtained from the NASA Level 1 and Atmosphere Archive and Distribution System (LAADS Web) [28]. A two-year average AOD raster data layer (10 km by 10 km grid) was calculated by overlaying the daily AOD layers and using a GIS cell statistics (mean) function. Data from both Terra and Aqua satellites were used. MODIS AOD data are not available every day due to cloud cover. If a pixel does not contain AOD data on a day, it was not included in temporal averaging calculation. Data for cold seasons (October to March) were not used in the calculation. Cloud cover, snow reflectivity, and diminished vertical mixing all reduce the accuracy of ground-level pollutant levels measured in winter. During warm seasons, vertical columns in the atmosphere are more integrated. AOD measures correlate best with ground-based monitoring in warm months, likely because of stronger boundary layer mixing during the warmer months [29]. Ideally, fine-mode optical depth should be used for the health effect assessment because fine mode particles which dominate urban/industrial pollution cause the most severe health problem. However, this paper did not use MODIS fine-mode AOD data product for health effect statistical analysis. Land-based measures of fine-mode fraction of AOD can only be used as a qualitative indicator of whether AOD values are dominated by natural or anthropogenic emissions [26].

### CIHD data

The study area covers US Census Regions 1 (Northeast), 2 (Midwest) and 3 (South) including 37 states and District of Columbia. CIHD (ICD-10 codes: I25.0–I25.6, I25.8, I25.9) data at the county level were extracted for the period from 2003 to 2004 from the National Center for Health Statistics Compressed Mortality File 1999–2005 in the CDC WONDER online database [30]. CIHD mortality count and population at risk was retrieved by county, race (White, Black or African American, Other race) and age (5 years interval from 1–4 to 85+). Aggregated CIHD mortality count and population at risk were also retrieved by race and age groups for the whole study area to be used as standard population in calculating CIHD mortality rate adjusting for race and age effects.

Race and age adjusted rate was calculated using indirect standardization [31] for each county. Rate adjustment is a technique for removing the effects of race and age from crude rates, so as to allow meaningful comparisons across populations with different underlying race and age structures. In the US, researchers normally use the year 2000 US decennial census data as a standard population to standardize rates. The current study covers only part of the US and a time period (2003–2004) different from the year 2000. The use of an internal standard population consisting of race-age-specific rates from a super-population containing the regions to compare would be better than an external standard based on individuals from an entirely separate population [32]. In the current study, to compare indirectly standardized rates between counties within the study area, data from the study area and period rather than from the whole US and the year 2000 was used as the standard population to obtain a standardized rate for each county.

The indirect standardization first calculated expected number of CIHD deaths for each county. The calculated death count is the number of cases that would be expected in the study population if people in the study population contracted the disease at the same rate as people in the standard population. Standardized mortality rates (SMRs) were calculated by dividing the observed count by the expected value. According to Curtin and Klein [33], one of the problems with rate adjustment is that rates based on small numbers of deaths will exhibit a large amount of random variation. In the aggregate CIHD mortality count and population data set, counts of twenty or less are flagged as statistically "unreliable". CIHD counts are "suppressed" when the data meets the criteria for confidentiality constraints. Counts for counties with census year populations of less than 100,000 are replaced with "suppressed" if the number of cases is five or less and the count is based on only one or two years of data. All counties with unreliable and suppressed data were not included in calculating standardized rates and spatial analysis and modelling thereafter. The number of counties in the study area is 2,506. With 200 counties that have unreliable or suppressed disease data being omitted, the number of data points (counties) for the statistical modelling is 2,306.

### Linking CIHD with AOD

To link the disease with air pollution, the 2003–2004 mean AOD raster grid was first resampled so that each 10 km by 10 km grid cell was subdivided into 10 by 10 smaller cells retaining the original AOD values. The purpose of the resampling procedure was to split the 10 km cell on the county boundary into separate parts for neighbouring counties to achieve higher accuracy of county average AOD calculation.

The resampled AOD grid was then overlaid with the map of CIHD rates. A GIS zonal statistical function was used to calculate the mean AOD value for each county. The mean AOD value was calculated by averaging AOD values of all cells whose centroids are within the county.

To assess the effect of aerosol particles on CIHD, exploratory spatial data analysis (ESDA) [34] and regression models were used to explore the association between CIHD SMR and AOD using GeoDa software [35]. ESDA methods involved using a bivariate global Moran's I statistic and local indicator of spatial association (LISA) [36]. Bivariate global Moran's I value determines the strength and direction of the relationship between two variables such as SMR and AOD in each county and measures the overall clustering. LISA provides information relating to the location of spatial clusters and outliers and the types of spatial correlation. Local statistics are important because the magnitude of spatial autocorrelation is not necessarily uniform over the study area [34]. Analysis results were presented as a Moran scatter plot and a cluster map in this analysis. Significance was tested by comparison to a reference distribution obtained by random permutations [34]. A random permutation procedure recalculates a statistic many times by reshuffling the data values among the map units to generate a reference distribution. The obtained statistic calculated based on the observed spatial pattern is then compared to this reference distribution and a pseudo significance level is computed. This analysis used 999 permutations to determine differences between spatial units. Spatial contiguity was assessed as Queen's contiguity which defines spatial neighbors as those areas with shared borders and vertexes. The Bivariate Moran's I is represented as the values of AOD averaged across all neighboring counties and plotted against SMR in each county. If the slope on the scatter plot is significantly different to zero then there is a relationship between SMR and AOD. The bivariate LISA cluster map incorporates information from the Moran scatter plot about the spatial relationships.

Three regression models were fitted to examine the relationship between SMR and AOD: ordinary least square (OLS), spatial lag, and spatial error. Fitting three models allows choice of a model with the best fit. Construction of the two spatial regression models could alleviate the problem of spatial autocorrelation within the data. Spatial autocorrelation is the propensity for data values closer to each other in space to be more similar. If spatial autocorrelation exists, the assumption of independent observations and errors of classical statistical models may be violated. Spatial regression methods capture spatial dependency in regression analysis, avoiding statistical problems such as unstable parameters and unreliable significance tests, as well as providing information on spatial relationships among the variables involved. The spatial lag model (also called Spatial Auto-Regressive model, or SAR) takes the form:(1)

and the spatial error model takes the form:(2)

where *y *is an (*N *× 1) vector of observations on the dependent variable taken at each of *N *locations, *X *is an (*N *× *k*) matrix of exogenous variables, *β *is an (k × 1) vector of parameters, and *ε *is an (*N *× 1) vector of disturbances, *ρ *is a scalar factor for the spatial lag term, *λ *is a scalar error parameter, *μ *is a spatially autocorrelated disturbance vector, and *W *is spatial weight. The spatial weight for this study was based on the first-order Queen's contiguity rule. If two counties share boundary or node, the weight is equal to 1, otherwise it is zero. The spatial weight matrix was then row standardized so that all columns sum to 1.

## Results

**Figure 1:**
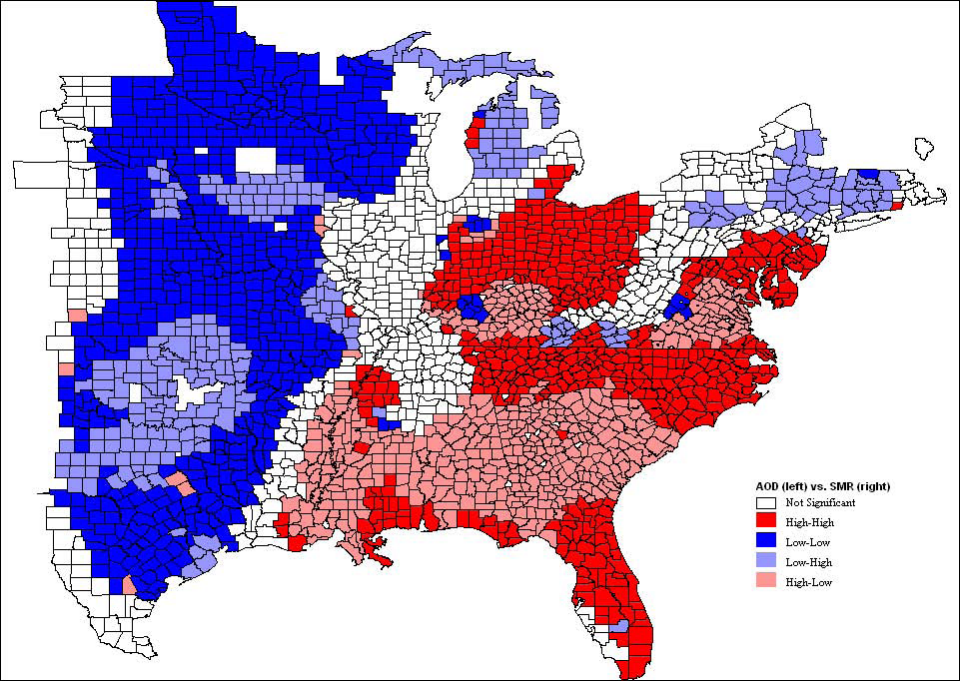
**Bivariate LISA cluster map**.

**Figure 2:**
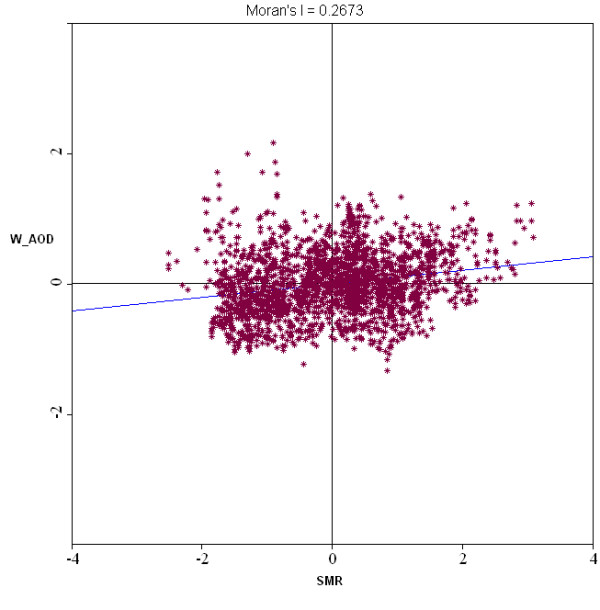
**Biavariate Moran's I scatter plot**.

The bivariate LISA cluster map is shown in Figure 1 (permutations = 999, *p *= 0.05). Note that this shows local patterns of spatial correlation at a county between SMR and the average AOD for its neighbors. Counties with significant spatial correlation are color coded by type of spatial autocorrelation. These four categories correspond to the four quadrants in the Moran scatter plot as shown in Figure 2, which shows a box plot for the distribution of the local Moran statistics across observations. The axes have been standardized such that the units correspond to standard deviations. The scatter plot figure has also been centered on the mean with the axes drawn such that the four quadrants are clearly shown. The high-high and low-low locations (positive local spatial correlation) represent spatial clusters, while the high-low and low-high locations (negative local spatial correlation) represent spatial outliers. It should be noted that the so-called spatial clusters shown on the LISA cluster map only refer to the core of the cluster [36]. The cluster is classified as such when the value at a location (either high or low) is more similar to its neighbours (as summarized by averaging the neighbouring values, the spatial lag) than would be the case under spatial randomness. Any location for which this is the case is labelled on the cluster map. However, the cluster itself likely extends to the neighbours of this location as well.

The global Moran's I value is 0.2673 (*p *= 0.001), indicating an overall positive spatial correlation of CIHD SMR and AOD. The overall positive correlation can also be seen on the LISA cluster map. The entire study area is dominated by spatial clusters: high AOD and high SMR in the east, low AOD and low SMR in the west. However, there are outlier counties in the southeast where there is high AOD but low SMR and in a small part of the west where AOD is low but SMR is high. These spatial outlier locations, especially areas with high SMR but low AOD, warrant further investigation to see if other factors dominate in effects on SMR and contribute to the attenuation of the air pollution effect. It must be noted that AOD does not differentiate between fine-mode and course-mode particles and that fine mode particles cause the most severe health problem. The spatially varying patterns of AOD-SMR association (i.e., both spatial clusters and outliers exist in the study area, although the clusters dominate) are partly due to the spatial varying relationship between AOD and PM_2.5_. Previous research has found that the association between AOD and PM_2.5 _depends on aerosol composition, vertical distribution, meteorology, terrain, and land use/cover, and therefore varies both temporally and spatially [25,37-39]. The algorithm to calculate AOD is based on dark surface pixels, contrast with surface, and assumptions in the model on the types of pollutants and the terrain. The lower correlations in general can be found in the more arid parts where the higher surface reflectance reduces contrasts. In our study area, the east region is humid and densely covered by vegetation, and hence AOD correlate very well PM_2.5_. Therefore, high AOD represents high PM_2.5 _in the east, where SMR is also high. Although the southeast has high AOD, our previous research [17] has found relatively lower fraction of fine-mode aerosols in the region, which might explain the relatively lower SMR rates. The low-AOD spatial outlier in the southern Midwest (Houston-Dallas metropolitan region) with high SMR could be due to high fraction of fine-mode particle near ground surface coming from the oil and gas industry activities.

Table 1 shows the OLS model result. CIHD SMR is significantly related to AOD (*β *= 0.9414, *p *= 0.000). Overall, CIHD morality rate increases with AOD. The low probability of the Jarque-Bera test score indicates non-normal distribution of the error term. The high probabilities of the three heteroskedasticity tests (Breusch-Pagan, Koenker-Bassett, and White) point to non-existence of heteroskedasticity. Moran's I score is positive and highly significant, indicating strong spatial autocorrelation of the residuals. Both simple Lagrange Multiplier (LM) tests of the lag and error are significant, indicating presence of spatial dependence. The robust LM measure for error is still significant, but the robust LM lag test becomes insignificant (*p *= 0.385), which means that when lagged dependent variable is present the error dependence disappears.

**Table 1:** Ordinary lease square regression

Model description						
	**Y**	**No. of variables**		**No. of observations**	**Degrees of freedom**	
	
	SMR	2		2306	2304	

**Model fit**						

	**R**^2^	**Adjusted R**^2^	**Log likelihood**	**F-statistic**	***p *(F-statistic)**	**AIC**
	
	0.0131	0.0127	-452.760	30.58	0.000	909.53

**Model estimation**						

	**Variable**	**Coefficient**		**Std. Error**	**t-Statistic**	***P***
	
	CONSTANT	0.7288		0.0284	25.689	0.000
	
	AOD	0.9414		0.1702	5.530	0.000

**Diagnostic tests**						

		**Tests**		**DF**	**Value**	***P***

**Normality of errors**		Jarque-Bera		2	264.404	0.000
		
**Heteroskedasticity**		Breusch-Pagan		1	0.014	0.906
		
		Koenker-Bassett		1	0.010	0.920
		
		White		2	0.650	0.723

**Spatial dependence**		Moran's I (error)		0.332	26.577	0.000
		
		Lagrange Multiplier (lag)		1	568.926	0.000
		
		Robust LM (lag)		1	0.754	0.385
		
		Lagrange Multiplier (error)		1	700.080	0.000
		
		Robust LM (error)		1	131.908	0.000
		
		Lagrange Multiplier (SARMA)		2	700.834	0.000

The spatial lag model (Table 2) shows the similar AOD effect (*β *= 0.5367, p < 0.001). The coefficient parameter (*ρ*) of the spatial lag term of CIHD age-race-adjusted rate (W_SMR) has a positive effect and is highly significant (*ρ *= 0.4962, *p *= 0.000), reflecting the spatial dependence inherent in the CIHD rates. SMR in a county is similar to the average of its neighbors. Including of the lag term has improved the general model fit, as indicated in higher values of R-squared and log likelihood. The high probability in the Breusch-Pagan test suggests that heteroskedasticity still does not exist after introducing the spatial lag term. In the likelihood ratio test of the spatial lag dependence, the result is significant. Although the introduction of spatial lag term improved the model fit, it did not make the spatial effects go away.

**Table 2:** Spatial lag regression model

Model description					
	**Y**	**No. of variables**	**No. of observations**	**Degrees of freedom**	
	
	SMR	3	2306	2303	

**Model fit**					

	**R**^2^		**Log likelihood**	**AIC**	
	
	0.2238		-235.357	476.713	

**Model estimation**					

	**Variable**	**Coefficient**	**Std. Error**	**t-Statistic**	***P***
	
	*ρ*	0.4962	0.0231	21.496	0.0000
	
	CONSTANT	0.3605	0.0304	11.859	0.0000
	
	AOD	0.5367	0.1522	3.527	0.0004

**Diagnostic tests**					

	**Tests**		**DF**	**Value**	***p***

**Heteroskedasticity**	Breusch-Pagan		1	0.156	0.6933

**Spatial dependence**	Likelihood Ratio		1	434.813	0.2745

In the spatial error model (Table 3), the coefficient on the spatially correlated errors (*λ*) has a positive effect and is highly significant (*λ *= 0.5678, *p *= 0.0000). As a result, the general model fit improved, as indicated in higher values of R-squared and log likelihood. Like the OLS and lag models, the effect of AOD remains positive and significant (*β *= 0.7774, p = 0.01). Similar to the lag model, the heteroskedasticity test remains insignificant, indicating non-existence of heteroskedasticity. The likelihood ratio test of spatial error dependence has an insignificant result. Allowing the error term to be spatially correlated not only improved the model fit, but also made part of the spatial effects go away. Since the spatial error model has the greatest R-squared and log likelihood values, it is the best of the three models linking CIHD with AOD.

**Table 3:** Spatial error regression model

Model description					
	**Y**	**No. of variables**	**No. of observations**	**Degrees of freedom**	
	
	SMR	2	2306	2304	

**Model fit**					

	**R**^2^		**Log likelihood**	**AIC**	
	
	0.2800		-201.834	407.669	

**Model estimation**					

	**Variable**	**Coefficient**	**Std. Error**	**t-Statistic**	***p***
	
	CONSTANT	0.7506	0.0509	14.759	0.0000
	
	AOD	0.7774	0.3022	2.573	0.0101
	
	*λ*	0.5678	0.0231	24.600	0.0000

**Diagnostic tests**					

	**Tests**		**DF**	**Value**	***p***

**Heteroskedasticity**	Breusch-Pagan		1	0.067	0.7954

**Spatial dependence**	Likelihood Ratio		1	501.856	0.0000

## Discussion

We found significant positive spatial correlation between age-race-standardized mortality rate of chronic ischemic heart disease and air pollution (indicated by satellite measured aerosol optical depth). There is an excess risk of CIHD mortality in areas with high air pollution levels. Several plausible mechanisms have been described to explain the association between air pollution and cardiovascular diseases, including enhanced coagulation and thrombosis, a propensity for arrhythmias, acute arterial vasoconstriction, systemic inflammatory responses, and the chronic promotion of atherosclerosis [40]. Fine aerosol particles tend to penetrate into the gas-exchange regions of the lung, and ultrafine particles are able to pass through the lungs to enter the blood circulation and affect other organs [41]. A study indicates that PM_2.5 _leads to high plaque deposits in arteries, causing vascular inflammation and atherosclerosis – a hardening of the arteries that reduces elasticity, which can lead to heart attacks and other cardiovascular problems [42,43].

There are several strengths in our study. First, ecological studies are particularly useful when disease data at individual level is not available and individual level of exposure is either difficult or impossible to obtain, or can be only measured imprecisely [44]. Ecological studies are more useful for generating and testing hypothesis [45]. The statistically significant positive association between CIHD mortality rate and aerosol particle pollution can be taken as indicative (though not necessarily causative) of a potential air pollution effect. The association would justify the need of further toxicological approach for investigating the biological mechanism of the adverse effect of particulate air pollution on heart. Second, the MODIS aerosol data lends itself to population-based exposure assessment and the ecological approach. Moderate resolution satellite image pixels share the same feature dimension with the disease data enumeration districts in that both pixels and county polygons are area features. This alleviates the non-straightforwardness and noncompliance of comparing discrete point monitoring data with area-aggregated disease data. Furthermore, a pixel value being an average of the variant radiometric measurements present in the pixel conforms to the need of deriving an average value of an air pollution indicator for each enumeration unit. Seamless remote sensing images better represent aggregate exposure than averaging ground measurements over a limited number of monitoring sites, and reduce the problem of spatially variant accuracy in geostatistical interpolation caused by uneven spatial distribution of monitors. Third, satellites offer a tremendous spatial coverage, which is suitable for regional air quality monitoring, pollution event warning, and environmental health studies. Although the spatial coverage for PM_2.5_monitoring system in the eastern US is adequate, most of the monitors are in urban areas. The MODIS aerosol retrieval algorithm uses the industrial/urban aerosol model in eastern US, Western Europe and eastern China [26] where a high positive correlation exists between AOD and PM_2.5_. There is a need to assess air quality for public health in these developed and densely populated regions. The finding of the association between aerosol data and the disease suggests that satellite aerosol data could be used as an indicator of fine particulate air pollution for public health effect assessment in regions/countries dominated by urban/industrial aerosols and without extensive air quality monitoring networks.

There are also several limitations to this study. First, while we adjusted effects of race and age, we did not consider other potential confounding factors, for example, smoking prevalence, cholesterol level, diabetes, blood pressure, gender, and socioeconomic status. The R^2 ^values of the regression models are low. Air pollution explains less than 30% of variance in CIHD SMR. Second, the use of aggregated data and therefore inferences based on the analysis cannot be directly transferred to the individual level. An inherent limitation of an ecological study is that it uses aggregate data and does not have the ability to incorporate individual information. Satellite measurements do not represent individual exposure. The population-based ecological analysis did not consider population dynamics, for example, population migration. According the US 2000 census data, nineteen percent of all moves made between 1999 and 2000 were from one state to another. People might have lived and exposed to air pollution in one county much earlier and later moved to another county where the first signs of the disease were diagnosed and where they died. Consideration of long-term population out-of-county movement is impossible without individual data. Third, it must be noted that there are limitations of AOD data. AOD is a quantitative measure of total column aerosol, which is the mass of aerosols within a measured column extending from Earth to the satellite sensor. Since AOD does not differentiate between fine-mode and course-mode particles, the relationship of AOD with PM_2.5 _as well as with disease does not necessarily hold for all regions. Human health is more related to fine aerosol particles from anthropogenic emissions than course particles. AOD does not specify the location of aerosols within a column and the AOD values do not necessarily represent ground conditions. Emerging Light Detection and Ranging (LIDAR) systems could provide vertical resolution for AOD and quantify pollutant levels on the ground. The best air quality assessments emerge from integrated datasets that include satellite data, ground-based measures and outputs from air quality models.

## Conclusion

The ecological study for counties in the eastern US found significant positive association between chronic ischemic heart disease mortality risk and air pollution. High risk of CIHD mortality was found in areas with elevated levels of outdoor particulate air pollution as indicated by satellite sensed aerosol optical depth. The evidence of raised CIHD mortality risk in high pollution areas would support targeting of policy interventions on such areas to reduce pollution levels. Remote sensing could help fill pervasive data gaps that impede efforts to study air pollution and protect public health. Remote sensing AOD data could be used as an alternative health-related indictor of air quality.

## List of abbreviations

AOD: aerosol optical depth; AQS: Air Quality System; CIHD: chronic ischemic heart disease; EOS: Earth Observation System; EPA: Environmental Protection Agency; ESDA: exploratory spatial data analysis; GWR: geographically weighted regression; ICD-10: International Classification of Disease, 10^th ^Revision; IHD: ischemic heart disease; LISA: local indicator of spatial association; LM: Lagrange Multiplier; MODIS: Moderate Resolution Imaging Spectrometer; NCEP: National Center for Environmental Prediction; OLS: ordinary least square; SAR: spatial auto-regressive model; SMR: standardized mortality rate.

## Competing interests

The authors declare that they have no competing interests.

## Authors' contributions

ZH conceived of the study, conducted the research and wrote the manuscript. All authors read and approved the final manuscript.
